# New York Dental Recorder

**Published:** 1848-01

**Authors:** 


*New York Dental Recorder.*—With the commencement of the second volume of this periodical, the editorship of the New York Dental Recorder, was transferred to Dr. C. C. Allen, an experienced dentist, and good writer. We have received numbers one, two and three of volume two, which contain a number of well written and interesting papers, and we are glad to perceive among the number, one from our London correspondent Dr. J. Robinson, copied from the American Journal of Dental Science.

It is said of the dental profession, that its members, as a body, are not reading men, that they have no taste for, nor pretensions to science, that they are satisfied with a mere meagre knowledge of the mechanical manipulations of the art. If this assertion has hitherto been true and even is at the present to some extent, we hope that not a single dentist will consent to remain under so disreputable an imputation any longer, but from this time henceforth resolve to avail himself of every means of information within his reach, and as publications of this sort are among the best, they should not be without them. The Dental Recorder is published in monthly numbers of twenty pages each at $2 per annum. We trust it will be well sustained. It may be had by application to Dr. C. C. Allen, 28 Warren street, New York.—*Balt. Ed.*

